# Prognostic Value of Morphological Characteristics and Immune Microenvironment in High-Grade Serous Cancer (HGSC)

**DOI:** 10.3390/cancers18142327

**Published:** 2026-07-19

**Authors:** Danijel Antonio Grubišić, Branka Petrić Miše, Toni Čeprnja, Vesna Telesmanić Dobrić, Vesna Čapkun, Snježana Tomić

**Affiliations:** 1 Department of Pathology, Forensic Medicine and Cytology, University Hospital Centre Split, 21000 Split, Croatia; toni.ceprnja@kbsplit.hr (T.Č.); snjezana.tomic@kbsplit.hr (S.T.); 2 Department of Pathology, School of Medicine, University of Split, 21000 Split, Croatia; branka.petric-mise@kbsplit.hr; 3 Department of Oncology, University Hospital Centre Split, University of Split, 21000 Split, Croatia; 4 Department of Oncology and Nuclear Medicine, Zadar General Hospital, 23000 Zadar, Croatia; vesna.telesmanic.dobric@bolnica-zadar.hr; 5 Department of Nuclear Medicine, University Hospital Centre Split, School of Medicine, University of Split, 21000 Split, Croatia; vesna.capkun@gmail.com

**Keywords:** high-grade serous ovarian cancer, SET morphology, tumor microenvironment, tumor-infiltrating lymphocytes, CD8-positive lymphocytes, PD-L1, lymphoid aggregates, progression-free survival, ovarian cancer immunology, immunophenotype

## Abstract

**Background/Objectives:** High-grade serous ovarian carcinoma (HGSC) is the most aggressive subtype of epithelial ovarian cancer and is characterized by marked heterogeneity of the tumor immune microenvironment. SET morphology has been associated with homologous recombination deficiency and BRCA1/2 mutations; however, its relationship with the immune microenvironment and progression-free survival (PFS) remains insufficiently understood. This study investigated the association between SET morphology, immune microenvironment characteristics, and PFS in patients with advanced-stage HGSC. **Methods:** A retrospective cohort of 305 patients with FIGO stage III–IV HGSC treated with primary surgery between 1996 and 2021 was analyzed. Histopathological assessment included evaluation of SET morphology, stromal and intraepithelial tumor-infiltrating lymphocytes (sTILs and itTILs), tumor immune phenotype, and lymphoid aggregates. Immunohistochemical analyses included CD8 and PD-L1 expression. Associations between SET morphology and immune parameters were evaluated using χ^2^ and logistic regression analyses. PFS was assessed using Kaplan–Meier analysis, log-rank testing, and Cox proportional hazards regression. **Results:** SET morphology was significantly associated with higher sTIL and itTIL levels, increased stromal and intraepithelial CD8^+^ T-cell infiltration, higher PD-L1 TPS and CPS, more frequent primary and secondary lymphoid aggregates, and a predominance of the inflamed immune phenotype (all *p* < 0.05). Despite these features of an immune-active tumor microenvironment, SET morphology, CD8^+^ T-cell density, PD-L1 expression, lymphoid aggregates, and immune phenotype were not independently associated with prolonged PFS. In contrast, age remained an independent prognostic factor, with patients older than 55 years having a 50% higher risk of disease progression than younger patients (HR = 1.5, 95% CI: 1.1–2.1; *p* = 0.012). Higher intraepithelial TIL levels (>10%) were independently associated with improved PFS (HR = 2.1, 95% CI: 1.0–4.4; *p* = 0.045). **Conclusions:** SET morphology identifies an immune-active subtype of HGSC characterized by increased immune infiltration and PD-L1 expression but does not independently predict prolonged PFS. The dissociation between immune cell abundance and clinical outcome suggests that immune cell functionality, rather than immune infiltration alone, may determine prognosis. Routine histopathological assessment of SET morphology may facilitate biological characterization of HGSC and provide a practical surrogate marker for future biomarker-driven studies evaluating immunotherapy and targeted treatment strategies.

## Simple Summary

High-grade serous ovarian carcinoma (HGSC) is the most aggressive subtype of ovarian cancer, and reliable biomarkers that reflect the tumor immune microenvironment are needed to improve patient stratification and guide future immunotherapy. In this study, we investigated whether the histological SET (solid, pseudoendometrioid, and transitional cell-like) growth pattern is associated with specific immune microenvironment features and clinical outcome. We found that tumors with SET morphology exhibited significantly higher stromal and intraepithelial tumor-infiltrating lymphocytes, increased CD8^+^ T-cell infiltration, higher PD-L1 expression, more frequent lymphoid aggregates, and predominantly inflamed immune phenotypes, indicating a more immunogenic tumor microenvironment. Despite these findings, neither SET morphology nor most immune parameters were associated with prolonged progression-free survival, suggesting that the presence of immune cells alone does not necessarily reflect effective antitumor immunity. Older age remained an independent predictor of shorter progression-free survival, while higher intraepithelial tumor-infiltrating lymphocyte levels were independently associated with improved progression-free survival. These findings suggest that routine histopathological assessment of SET morphology may provide a practical surrogate marker of tumor immunogenicity and support further studies integrating histopathology with functional immune and molecular biomarkers to improve patient selection for targeted and immunotherapeutic approaches.

## 1. Introduction

High-grade serous ovarian cancer (HGSC) is the most common and most aggressive histological subtype of epithelial ovarian cancer, accounting for approximately 70–80% of ovarian cancer deaths [1].

About 70% of ovarian cancers are diagnosed at an advanced stage when they have already spread beyond the ovaries. Unfortunately, only 30% of women with these cancers survive for 5 years or more [2]. Significant efforts have been made to diagnose HGSC earlier, traditionally using more frequent transvaginal ultrasonography with CA-125 as a serum marker. However, up to now, there is no definitive screening method that reduces HGSC mortality [3].

A distinct morphological pattern of HGSC is the SET pattern, which is characterized by architectural features that differ from the conventional papillary or glandular morphology of HGSC. The SET pattern comprises pseudoendometrioid architecture that mimics endometrioid carcinoma but retains high-grade nuclear atypia, a solid growth pattern composed of large sheets of tumor cells with scant stroma, and a transitional cell-like pattern resembling urothelial carcinoma. Initially described by Soslow et al. in association with BRCA1/2-mutated ovarian carcinomas, subsequent studies, including that of D’Angelo et al., have demonstrated that SET morphology is not merely a histological description but also has important biological and clinical implications, particularly in the context of personalized treatment and the use of targeted therapies [4,5].

The main treatment for most ovarian cancer patients, including HGSC, involves surgery followed by chemotherapy [6]. Currently, the standard first-line chemotherapy regimen consists of carboplatin in combination with paclitaxel [7]. However, new treatment options, such as bevacizumab and PARP inhibitors for platinum-sensitive disease, as well as folate receptor α-targeted therapies and immune checkpoint inhibitors (ICIs) for platinum-resistant ovarian cancer, have been introduced into the treatment of ovarian cancer in combination with chemotherapy [8,9]. Analysis of tumor immune microenvironment (TME) may help to identify which patients will respond well to ICI.

Tumor-infiltrating lymphocytes (TILs) are defined as lymphocytes that directly target and/or surround tumor cells. There is growing evidence that TILs have a significant impact on the prognosis and tumor response to various treatment regimens in different cancers [10].

In tumor pathology, TILs are commonly categorized based on their spatial distribution in relation to tumor cells. Accordingly, TILs are typically divided into stromal TILs (sTILs), located in the tumor stroma surrounding malignant cell nests, and intratumoral or intraepithelial TILs (itTILs), which are found within tumor cell nests in direct contact with malignant cells [11]. CD8 immunohistochemistry is frequently used to quantify tumor-infiltrating cytotoxic T lymphocytes, including both stromal (sTIL) and intratumoral/intraepithelial TILs (itTILs), as CD8^+^ T cells represent a major effector population involved in anti-tumor immune responses [11,12]. Lymphoid cell clusters within tumors can organize into tertiary lymphoid structures (TLS), which are similar to secondary lymphoid organs. They are divided into primary lymphoid aggregates, dense clusters of lymphocytes without germinal centers, and secondary aggregates, which contain organized germinal centers with proliferating B cells and supporting follicular dendritic cells [13]. Tumor PD-L1 expression is widely used as a predictive biomarker for the response to immune checkpoint inhibitors in various type of cancers [14].

The tumor immune microenviroment (TME) can be classified into three distinct types based on histopathology and immunology: inflamed, excluded, and desert. These immune phenotypes are associated with prognosis in various types of tumors [15]. Traditionally, HGSC has been regarded as a relatively immunologically “cold” tumor because of its immunosuppressive tumor microenvironment and the limited efficacy of immunotherapy. Nevertheless, accumulating evidence suggests that HGSC is among the tumors in which anti-tumor immune response is strongly linked to improved survival. In several clinical trials, a subset of patients has demonstrated durable responses, pointing to the presence of an immunogenic subgroup of HGSC patients who may derive greater benefit from immunotherapeutic approaches [16,17].

It has been over a decade since the FDA approved ipilimumab as the first checkpoint inhibitor in metastatic melanoma [18]. Cancer immunotherapy has become a treatment option for various solid and hematological malignancies. The effectiveness of immunotherapy differs considerably, even among patients diagnosed with the same type of tumor [19]. The phase II study by How and al. assessed the addition of pembrolizumab to standard first-line chemotherapy in patients with advanced ovarian cancer and reported promising clinical activity with an acceptable safety profile. The translational analyses also indicated that patients with higher PD-L1 expression and a more immune-active tumor microenvironment may benefit more from this combined chemo-immunotherapy approach [20]. Also, a recent phase III randomized, double-blind study, KEYNOTE-B96 (NCT05116189), demonstrated that the addition of pembrolizumab to standard paclitaxel-based chemotherapy, with or without bevacizumab, significantly improves outcomes in patients with platinum-resistant epithelial ovarian cancer. Compared with the control group, a statistically significant prolongation of progression-free survival (PFS) and overall survival (OS) was observed, particularly in patients with PD-L1-positive tumors (CPS ≥ 1) [21]. Therefore, on 10 February 2026, the US Food and Drug Administration (FDA) approved pembrolizumab ((Keytruda; Merck & Co., Inc., Rahway, NJ, USA)), as well as pembrolizumab combined with berahyaluronidase alfa-pmph (Keytruda Qlex; Merck & Co., Inc., Rahway, NJ, USA), in combination with paclitaxel with or without bevacizumab, for the treatment of adult patients with platinum-resistant epithelial ovarian, fallopian tube, or primary peritoneal carcinoma whose tumors express PD-L1 (CPS ≥ 1) as determined by an FDA-authorized test, following one or two prior systemic treatment regimens [22].

In this study, we analyzed the association between SET morphology and components of the tumor immune microenvironment (TILs, CD8 TILs, PD-L1, lymphatic aggregates (LAs) and progression-free survival (PFS)) in patients with advanced-stage HGSC, as well as their potential impact on PFS.

## 2. Materials and Methods

### 2.1. Patients

Case records of patients with advanced-stage HGSC (FIGO stage III and IV) treated at the University Hospital Centre Split and General Hospital Zadar, Croatia, who underwent primary surgery between 18 January 1996 and 16 December 2021, were retrospectively reviewed. The last included patient underwent surgery on 16 December 2021, received the final cycle of adjuvant chemotherapy on 10 June 2022, and was followed until the study cut-off date of 1 January 2023. Based on pathology reports, this study included 305 HGSC patients with available tissue blocks and clinical information. Of the total 305 patients, 262 (86%) were from University Hospital of Split and 43 (14%) were from General Hospital Zadar.

The median age of the patients at the time of diagnosis of HGSC was 61 years. The youngest patient was 26 years old, and the oldest was 88 years old.

Progression-free survival (PFS) was defined as the time from surgery to documented disease progression according to RECIST criteria or death from any cause, whichever occurs first [23].

All patients included in the study initially underwent primary surgery. A total of 191 out of the 305 patients (62.6%) received chemotherapy based on platinum compounds, and those patients were included in PFS analysis. Patients who received first-line maintenance therapy with PARP inhibitors, either as monotherapy or in combination with bevacizumab, as well as patients treated with non-platinum chemotherapy because of platinum intolerance or those who received supportive care only due to age and/or significant comorbidities, were excluded from the PFS analysis.

Patients were followed according to the Croatian national clinical guidelines for ovarian cancer, with scheduled follow-up visits every 3–4 months during the first 2 years after primary treatment, every 6 months during years 3–5, and annually thereafter. Follow-up included medical history, physical and gynecological examinations with pelvic ultrasonography, routine laboratory tests, and serum CA-125 measurement in patients with initially elevated CA-125 levels. Computed tomography (CT) of the chest and abdomen was performed only when clinically indicated, such as in patients with residual disease after surgery or when recurrence was suspected based on clinical findings or rising CA-125 levels [24].

All histopathological evaluations, including assessment of SET morphology, tumor-infiltrating lymphocytes (TILs), and the immune phenotype of the tumor microenvironment, were performed independently by three experienced pathologists (D.A.G., T.Č., and S.T) who were blinded to all clinical and outcome data. Although a formal interobserver agreement analysis was not performed, all discrepant cases were subsequently reviewed jointly, and the final classification was established by consensus among the three pathologists. The histological type of ovarian cancer was determined following the last World Health Organization (WHO) [25]. This study received approval from the Ethics Committee of the Clinical Hospital Centre Split and the School of Medicine, University of Split, Croatia. It was conducted in accordance with the World Health Organization’s Declaration of Helsinki in 1975, as revised in 2013, and the International Conference on Harmonization Guidelines on Good Clinical Practice [26,27]. The patients’ anonymity was fully protected.

### 2.2. Histopathology and Immunohistochemistry

Upon reviewing the hematoxylin-eosin-stained (H&E) archival slides, we have analyzed the morphological and immunological characteristics of the tumor and its microenvironment, which includes confirmation of diagnosis of HGSC, presence of SET morphology, quantity of stromal and intratumoral TILs, and presence of lymphatic aggregates (LAs).

**Figure 1:**
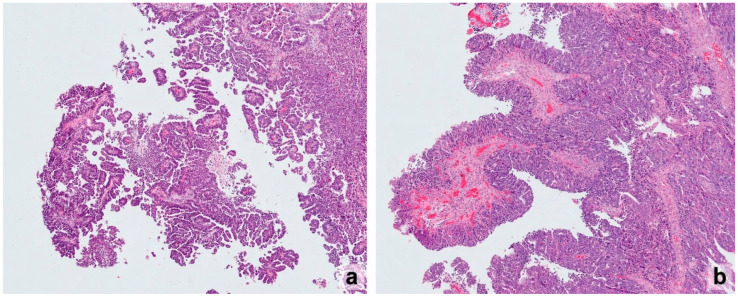
Tumor morphology (H&E staining): (**a**) classical high-grade serous carcinoma, characterized by numerous papillary structures; (**b**) SET-pattern morphology of high-grade serous carcinoma, demonstrating transitional cell-like growth.

The tumors exhibiting specific architectural patterns (pseudoendometrioid, solid and transitional-cell like) were classified as SET. Representative examples of the classical HGSC morphology and SET morphology are shown in Figure 1.

To address tumor variability, we examined whole tissue sections to identify stromal and intratumoral TILs, avoiding necrotic areas and technical errors. Since there are no standardized guidelines for assessing stromal and intratumoral TILs in ovarian tumors, we followed the recommendations for counting of sTIL of the International TIL Working Group for breast cancers [11]. We measured the finding of stromal lymphocytes (sTIL) as a percentage (%) of the stromal area infiltrated by immune cells within the tumor and at its invasive border. Although the recommendations issued by the International TIL Working Group are not as strictly defined for the assessment of itTILs, in our study, itTILs were quantified by estimating the percentage of tumor epithelium infiltrated by lymphocytes.

**Figure 2:**
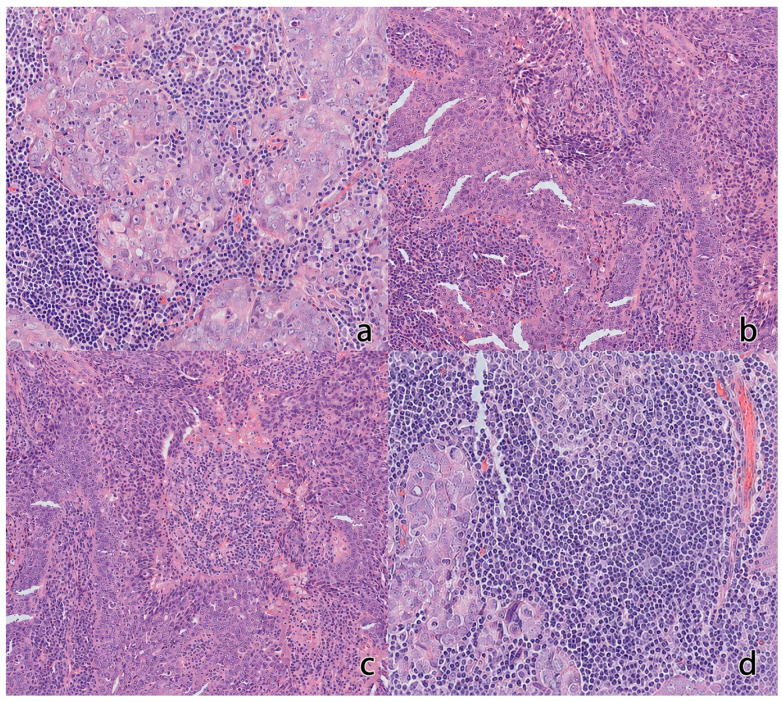
Tumor microenvironment (H&E staining): (**a**) high levels of intratumoral tumor-infiltrating lymphocytes (itTILs) in direct contact with tumor cells; (**b**) abundant stromal tumor-infiltrating lymphocytes (sTILs) occupying the majority of the stromal compartment at the periphery and between tumor cell islands; (**c**) primary lymphoid aggregate formation, clearly distinguishable from the surrounding stromal TILs; (**d**) secondary lymphoid aggregate with a germinal center located at the invasive tumor front.

We analyzed the presence of lymphoid aggregates (LAs) both within the tumor and within 5 mm from the outer edge of the tumor. We further classified them as primary or secondary based on the presence or absence of germinal centers (GCs). LAs without GCs are considered primary, while those with GCs are considered secondary [28]. Examples of the evaluated tumor microenvironment features are shown in Figure 2.

The immune response in the TME was assessed by examining HE slides. It was categorized as follows: (1) Desert—indicating a desmoplastic stroma with very few or no inflammatory cells, (2) Excluded—showing inflammatory cells in the stroma but not within the tumor, and (3) Inflamed—representing a presence of inflammatory cells both in the stroma and within the tumor tissue [29].

In the next step, we stained representative sections of the tumor tissue using the immunohistochemical method for CD8 and PD-L1 staining. This was carried out according to the standard protocol at the Laboratory for Immunohistochemistry, Department of Pathology, Forensic Medicine, and Cytology, University Hospital Centre Split.

The paraffin blocks containing tumor samples were additionally sectioned into 4 μm thick sections. The sections underwent standard pre-analysis procedures in the Ventana BenchMark ULTRA staining system (Ventana Medical Systems, Inc., Tucson, AZ, USA),including deparaffinization, rehydration, detection of antigenic epitopes, blockade of endogenous peroxidase activity, and immunohistochemical staining. The antibodies used in this study were PD-L1 (PD-L1 IHC 22C3 pharmDx (Dako, Agilent Technologies, Glostrup, Denmark); dilution 1:50) and CD8 (rabbit monoclonal antibody, clone SP239; dilution 1:100; Ventana Medical Systems, Inc., Tucson, AZ, USA). The PD-L1 Dako 22C3 was visualized using the OptiView DAB IHC Detection Kit (Ventana Medical Systems, Inc., Tucson, AZ, USA), while the CD8 was visualized using ultraView Universal DAB Detection Kit (Ventana Medical Systems, Inc., Tucson, AZ, USA).

Lymph node samples were utilized as positive controls for CD8 staining. The results were independently analyzed by three observers who were unaware of the clinical data.

The percentage of sTILs and itTILs that were CD8 positive was determined using immunohistochemistry. For each tumor sample, the highest number of CD8-positive sTIL and itTILs at the highest magnification of the microscope (×400) was counted. The specific area for the measurement was identified by examining the entire preparation at low magnification (×4) and locating the “hot spot” with the highest density of TILs.

**Figure 3:**
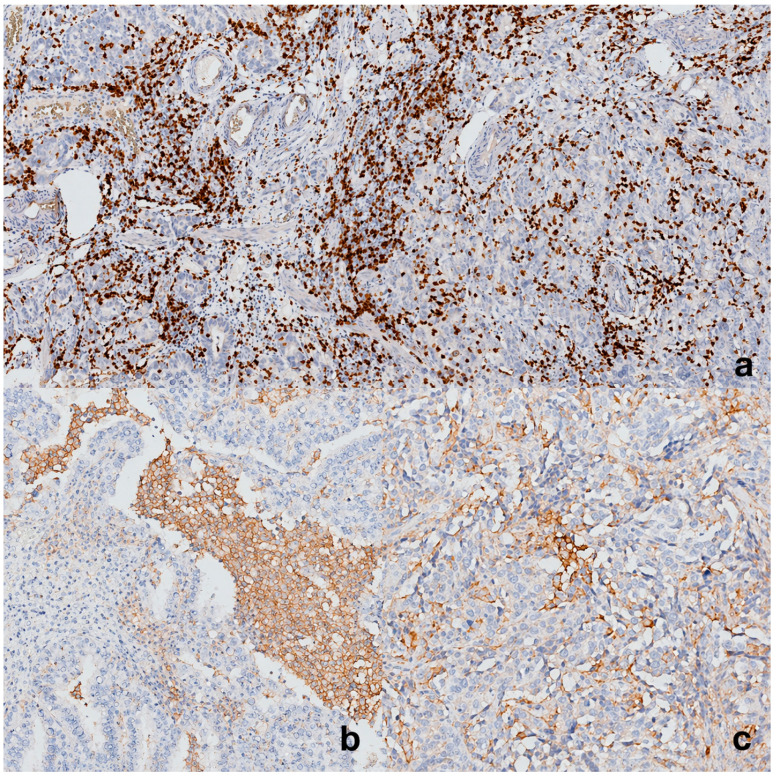
(**a**) Immunohistochemical staining with anti-CD8 antibody highlighting both stromal and intraepithelial tumor-infiltrating lymphocytes (TILs); (**b**) strong membranous and cytoplasmic staining of intratumoral lymphocytes and macrophages (DAKO 22C3 assay); (**c**) focal membranous staining of tumor cells with membranous and cytoplasmic staining of surrounding inflammatory cells (DAKO 22C3 assay).

For accurate and precise interpretation of PD-L1 results, tonsillar tissue was used as control. The results were read by two independent observers who did not know the clinical data. The immunohistochemical staining patterns evaluated in this study are illustrated in Figure 3a–c.

PD-L1 TPS (eng. PD-L1 tumor proportion score) is considered positive if any intensity of membranous staining is visible in ≥1% of tumor cells. It is calculated by dividing the number of positive tumor cells by the total number of viable tumor cells and then multiplying the result by 100 [30].

PD-L1 CPS (eng. PD-L1 combined proportion score) is considered positive if staining of any intensity is visible in ≥1 of tumor cells (membranous staining) and immune cells (lymphocytes and macrophages—membranous or/and cytoplasmatic staining). It is calculated by dividing the number of positive tumor cells, lymphocytes, and macrophages by the total number of viable tumor cells and then multiplying by 100 [31].

### 2.3. Statistical Analysis

The data was recorded in Microsoft Excel for Windows 2007 (Microsoft Corporation, Redmond, Washington, USA), and Statistical Package for the Social Sciences (SPSS) software (version 20 for Windows; SPSS Inc., Chicago, Illinois, USA) was used to calculate statistical significance. The results are presented in tables and interpreted at a significance level of *p* < 0.05.

We used the χ^2^ test and logistic regression to compare categorical variables. Cox regression analysis was used to determine the association of all investigated variables with the length of progression-free survival (PFS) of patients in months. In the analysis of survival according to all variables, we used the Kaplan–Meier survival curve and the log-rank test.

## 3. Results

### 3.1. Correlation of SET Morphology with Age of the Patients and Immune Microenvironment

Study cohort (*n* = 305) included 246 (81%) carcinomas without SET morphology and 57 (19%) carcinomas with SET morphology (Table 1).

We did not observe significant correlation between SET morphology and age groups (*p* = 0.370).

When we categorize the results of sTILs into three groups (<1, 1–10, >10), we found that tumors with SET morphology had higher levels of sTILs compared to tumors without SET morphology (*p* < 0.001).

When we categorize the results of itTILs into three groups (<1, 1–10, >10), we found a statistically significant difference in the itTIL groups in relation to the SET morphology (*p* < 0.001).

When we categorize results of itTILs into two groups (≤1, >1), a substantially greater proportion of SET tumors showed elevated itTIL levels >1 (72% vs. 32%), whereas itTIL levels ≤1 were considerably less frequent in the SET group (28% vs. 68%) (*p* < 0.001).

Based on ROC analysis (*p* = 0.005), we categorized the number of sCD8 lymphocytes in two groups: sCD8 ≤ 38.5 (*n* = 142) and sCD8 > 38.5 (*n* = 160). A significant association was observed between CD8^+^ T-cell number and SET morphology (*p* = 0.006). Based on ROC analysis (*p* < 0.001), we categorized the number of itCD8 lymphocytes in two groups: itCD8 ≤ 72,5 (*n* = 153) and itCD8 > 72,5 (*n* = 149). Similarly, higher intratumoral CD8^+^ T-cell number was more commonly observed in tumors with SET morphology (*p* = 0.001).

Tumors with SET morphology demonstrated significantly higher PD-L1 expression compared to tumors without SET morphology, as assessed by both TPS ≥ 1 and CPS ≥ 1 (*p* < 0.001).

Tumors with SET morphology demonstrated a higher frequency of lymphoid aggregates (LAs) compared to tumors without SET morphology, both in primary and secondary settings. Primary lymphoid aggregates were observed in 32% of SET tumors, compared to 17% of non-SET tumors (*p* = 0.017). Similarly, secondary lymphoid aggregates were more frequently identified in SET tumors (12% vs. 3%) (*p* = 0.007).

The concurrent presence of both primary and secondary LAs was substantially more frequent in tumors with SET morphology (12.3% vs. 3%) (*p* = 0.002).

Tumors with SET morphology demonstrated a markedly different tumor immunophenotype distribution compared to tumors without SET morphology, with a predominance of the inflamed phenotype. In contrast, immune-desert and immune-excluded phenotypes were substantially less frequent in the SET group (*p* > 0.001).

### 3.2. Prognostic Significance

The average progression-free survival (PFS) was 20.3 months (SE = 1.7; 95% CI: 17–24). The median of PFS was 13 months (SE = 0.7; 95% CI: 12–14). Patient age was significantly associated with PFS (χ^2^ = 7.8, *p* = 0.049).

As no significant differences in PFS were observed among patients aged 56–61, 62–69, and >69 years (LR = 0.084, *p* = 0.959), these groups were combined into a single ≥56 age category. Compared with patients aged 26–55 years, those aged ≥56 years had an 11-month shorter median PFS (17 months; LR = 7.8, *p* = 0.005) and a 1.5-fold higher risk of disease progression or recurrence (HR = 1.5, *p* = 0.007).

This study found no significant difference in PFS between three itTILs categories (*p* = 0.102). There was also no statistically significant difference in PFS between groups 1 and 2 (itTIL < 1, 1–10) (*p* = 0.736), so they were combined into one group—itTIL ≤ 10. The average PFS in the itTIL > 10 group was 22 months longer than in the itTIL ≤ 10 group (*p* = 0.034), and the risk of disease recurrence was found to be 2 times higher in the itTIL ≤ 10 group.

Based on the statistical analysis, there is no significant difference in PFS based on the presence of SET morphology (*p* = 0.105), number of sCD8 (*p* = 0.784), itCD8 (*p* = 0.745), PD-L1 TPS (*p* = 0.14), or PD-L1 CPS (*p* = 0.797). The association between progression-free survival (PFS) and the analyzed parameters is presented in Table 2 and Table 3.

Similarly, no significant difference was found based on presence of primary LAs (*p* = 0.706) or secondary LAs (*p* = 0.600) when dividing primary LAs and secondary LAs into three categories (*p* = 0.857) or tumor immunophenotype (*p* = 0.359).

To evaluate the association between progression-free survival (PFS) and the investigated variables, a multivariable Cox proportional hazards regression analysis was performed. The independent variables included age, sTILs, itTILs, and PD-L1 tumor proportion score (TPS). Although several variables were evaluated in the univariable Cox regression analysis, not all were included in the multivariable model because of substantial clinical correlation between individual parameters in order to minimize multicollinearity and improve model interpretability. The results of the multivariable Cox proportional hazards regression analysis are presented in Table 4.

Multivariable Cox regression analysis identified both age and intraepithelial tumor-infiltrating lymphocytes (itTILs) as independent prognostic factors for PFS. Compared with patients aged 55 years or younger, those older than 55 years had a higher risk of disease progression (HR = 1.50, *p* = 0.012). Similarly, patients with itTILs < 10% had a significantly higher risk of disease progression than those with itTILs > 10% (HR = 2.1, *p* = 0.045).

## 4. Discussion

HGSC is a histological subtype of ovarian cancer in which SET morphology is often associated with BRCA mutations and/or homologous recombination deficiency, a molecular phenotype often associated with increased tumor immunogenicity and higher levels of tumor-infiltrating lymphocytes [4,32,33].

The presence of intratumoral T cells in some publications has been shown to correlate with improved clinical outcomes in ovarian cancer [34]. Cytotoxic CD8+ T lymphocytes represent key effectors of antitumor immunity, as they recognize tumor antigens presented on MHC class I molecules and can directly induce tumor cell death [35]. In addition, regulatory T cells within the tumor microenvironment can suppress cytotoxic T-cell activity and contribute to tumor immune evasion [36].

In our study, tumors with sTIL > 10 are more frequent in the SET group. Although we did not identify studies that directly evaluate SET morphology in relation to sTILs as a distinct and separately quantified component of the overall TIL population, numerous reports have linked BRCA mutations—frequently observed in tumors with SET morphology—to increased intercellular TILs [5].

Assessment of sTILs shows a high level of interobserver reproducibility, whereas itTILs are more difficult to consistently and reliably identify in routine diagnostic practice without the support of additional immunohistochemical staining [28]. ItTILs are detected less frequently and are generally present at lower levels compared with sTILs [11]. In our study, itTILs levels > 1 were observed in 72% of tumors with SET morphology (*p* < 0.001). There are no published studies that precisely link histologically determined itTILs with SET characteristics except a study by Khashaba et al., who demonstrated that HGSCs with SET morphology more frequently have CD8+ lymphocytes in the intraepithelial component of the tumor [37]. In our study, we demonstrated a significant association between SET morphology and increased stromal and intratumoral number of CD8^+^ T-cell.

Importance of TILs for tumor immunogenicity was demonstrated in a study of Sato et al. in which intraepithelial CD8^+^ TILs and a high CD8^+^/Treg ratio correlated with significantly improved patient survival, indicating the presence of an effective antitumor immune response [38]. Webb et al. reported that the prognostic significance of CD8+ tumor-infiltrating lymphocytes is more pronounced when these cells are located within the tumor epithelium (itTILs) rather than in the surrounding stromal compartment. This observation suggests that the direct physical interaction between cytotoxic lymphocytes and malignant epithelial cells facilitates a more effective antitumor immune response [39]. However, the presence of intraepithelial CD8^+^ lymphocytes does not provide information regarding their functional state. Increasing evidence indicates that many tumor-infiltrating CD8^+^ T cells in HGSC express multiple inhibitory receptors, including PD-1, TIM-3, TIGIT, and LAG-3, consistent with an exhausted phenotype associated with impaired antitumor activity [40,41].

In addition to the previously mentioned regulatory T cells, tumor cells employ various mechanisms to evade immune surveillance and antitumor immune responses. One of the key mechanisms involves the overexpression of the PD-L1 protein on the surface of tumor cells, which binds to the programmed cell death protein 1 (PD-1) receptor expressed on T lymphocytes, thereby inhibiting their effector function and enabling tumor cells to escape immune-mediated destruction [42,43]. In the literature, we did not find any studies that explicitly link SET morphology in HGSC with PD-L1 expression; therefore, our study is the first to demonstrate that using the tumor proportion score (TPS), PD-L1 positivity (≥1%) was observed in 35% of SET tumors compared with 10% of non-SET tumors (*p* < 0.001). Using combined positive score (CPS), PD-L1 positivity (≥1) was present in 49% of tumors with SET morphology and in 23% of tumors without SET morphology (*p* < 0.001).

But, increased immune infiltration should not be interpreted as synonymous with an effective antitumor immune response. SET tumors in our cohort demonstrated significantly higher levels of CD8^+^ T cells but also PD-L1 expression. These findings may also reflect a state of adaptive immune resistance rather than productive immune activation. Persistent antigen exposure within HGSC promotes progressive T-cell dysfunction characterized by exhaustion, impaired cytokine production, and reduced cytotoxic capacity despite the continued presence of tumor-infiltrating lymphocytes. In this context, PD-L1 upregulation may represent an adaptive mechanism by which tumor cells counteract ongoing immune pressure and suppress T-cell effector function. Consequently, abundant CD8^+^ lymphocytic infiltration alone does not necessarily predict improved clinical outcome, particularly in the absence of functional immune profiling [44].

B cells represent an important component of the adaptive immune response and contribute to tumor immunogenicity through multiple mechanisms [45]. Within tumors, B lymphocytes can promote antitumor immunity by producing tumor-specific antibodies and facilitating antigen presentation to T cells [46]. In addition, B cells are key components of tertiary lymphoid structures, which have been associated with enhanced immune activation and improved responses to immunotherapy in several cancers [47,48,49]. The presence of LAs has been associated with favorable clinical outcomes in colorectal carcinoma and TNBC; however, their biological and prognostic significance in HGSC has not yet been comprehensively or conclusively elucidated [50,51,52].

Tumors with SET morphology exhibited a 4.4-fold higher proportion of both primary and secondary LAs than tumors with classical morphology (*p* = 0.004). However, it is important to note that the number of samples in which this finding was observed was limited. To our knowledge, there are no published studies that have specifically investigated the association between primary and secondary lymphoid aggregates and SET morphology.

Tumors exhibiting SET morphology demonstrated a markedly different immune phenotype compared with tumors without SET morphology. The inflamed tumor immunophenotype was substantially more frequent among tumors with SET morphology, whereas the immune-desert and immune-excluded phenotypes were predominantly observed in tumors without SET morphology (*p* < 0.001). In the literature, there are no studies that directly support these results.

In our study, TILs were present in all samples and predominantly were located in the stromal regions of the tumor areas, which is consistent with the research of Miceska et al. [53]. In many tumors, a high level of sTILs is related with high itTILs. However, this is not a rule—it is possible to have high sTIL levels without significant itTILs, or vice versa, depending on the tumor type and the specific immune dynamics. sTILs serve as a preparatory/stromal reservoir of cells, whereas itTILs are directly engaged in the fight against the tumor [11].

In our cohort, stromal TILs (sTILs) were observed in <1% of the stromal area in 10.82% of cases, in 1–10% of the stromal area in 62.62% of cases, and in >10% of the stromal area in 26.56% of cases. This result is consistent with the study by Hwang et al., who reported that 23% of EOC were included in the high stromal TILs group, with more than 10% sTILs [54]. Several researchers have assessed the presence of sTILs in ovarian carcinomas; however, different percentage cut-offs were used to define high sTILs infiltration, and some cohorts included other histological subtypes of ovarian carcinoma as well as samples previously treated with neoadjuvant chemotherapy; therefore, the results are impossible to compare.

We found that in 13.11% of cases, itTILs were in close contact with 10% or more tumor cells. Farrag et al. had an almost three-time higher percent of tumors with high itCD8 expression. They have used the same cut off of 10%, but they had only 54 tumor specimens in their research [55].

Results from the single-arm phase II trial (NCT02520154) demonstrated that the addition of pembrolizumab to frontline carboplatin–paclitaxel chemotherapy for advanced epithelial ovarian cancer was feasible without compromising the delivery of standard treatment. Notably, patients with higher PD-L1 expression (CPS ≥ 10) demonstrated more favorable survival outcomes, suggesting that PD-L1 expression may represent a potential predictive biomarker for response to PD-1 blockade [20]. More recently, the phase III ENGOT-ov65/KEYNOTE-B96 trial demonstrated significant improvements in progression-free and overall survival with pembrolizumab plus weekly paclitaxel, with or without bevacizumab, in patients with recurrent platinum-resistant epithelial ovarian cancer, particularly in those with PD-L1 CPS ≥ 1. However, these findings were obtained in the recurrent platinum-resistant setting rather than in treatment-naïve HGSC and therefore should not be directly extrapolated to the primary treatment setting [21].

In our study, PD-L1 TPS was ≥1 in 15% cases. Pizzaro et al. found only 8.1% HGSC which expressed PD-L1 in ≥1% of cells (TPS ≥ 1) [56]. The main difference between our two studies is that Pizzaro included only early-stage HGSC in his research, but it is widely recognized that PD-L1 expression is often higher in advanced stages of tumors than in early stages [57]. In their study of HGSC, Chen et al. reported that PD-L1 expression was observed in 21% of cases when applying a 1% TPS cut-off [58].

In our study, the PD-L1 CPS was ≥1 in 27% cases. Chen et al., in their study using the combined positive score (CPS) with a cut-off of 1, found that a positive PD-L1 expression was present in 48% of HGSC [58]. However, it is important to emphasize that the PD-L1 clone used in this study was not specified. In the study by Aronson et al., prior to the initiation of neoadjuvant therapy, 11 of 33 tumors (33.3%) demonstrated a PD-L1 CPS ≥ 10 [59].

Better understanding the spatial and functional units of immune populations within the tumor can advance the development of combined therapeutic approaches (e.g., checkpoint blockers + modulation of the TME), which is the subject of intensive research [60,61,62].

In our study, presence of SET morphology was not significantly associated with PFS. The available literature on this issue is limited and not entirely consistent. Uner et al. reported that SET-predominant HGSC was associated with more favorable survival outcomes and longer time to progression (TTP) [63]. However, the prognostic effect of SET morphology may not be independent. Ritterhouse et al. showed that non-classic morphology, including SET-like features, was associated with homologous recombination pathway mutations and improved PFS, suggesting that the survival advantage may be driven more by underlying molecular alterations than by morphology itself [64].

Taken together, our findings suggest that the lack of a significant PFS benefit despite the markedly inflamed phenotype observed in SET tumors may reflect the biological distinction between immune infiltration and immune function. Histological assessment and conventional immunohistochemistry quantify the presence of immune cells but cannot determine whether these lymphocytes retain cytotoxic activity or have entered a dysfunctional exhausted state. Likewise, the increased PD-L1 expression observed in SET tumors may reflect adaptive immune resistance induced by interferon-γ signaling rather than effective immune-mediated tumor control. Therefore, the coexistence of abundant CD8^+^ T cells and elevated PD-L1 expression, without additional analysis of CD8+ subtypes, may explain why enhanced immune infiltration was not accompanied by prolonged PFS in our cohort.

In the present study, a statistically significant difference in PFS across age groups (26–55, ≥56) was observed, with younger patients demonstrating a more favorable outcome compared to older age categories (*p* = 0.005). In the multivariable Cox regression analysis, age remained an independent predictor of shorter PFS (*p* = 0.012). However, it should be emphasized that these findings should be interpreted with caution, particularly in light of potential confounding clinicopathological factors. Our results are consistent with previous studies suggesting an association between younger age and improved survival outcomes in HGSC. For instance, Deng et al. reported that older patients exhibited significantly shorter PFS and overall survival compared to younger patients, with age remaining an independent prognostic factor in multivariate analysis [65]. Similarly, Hoppenot et al. highlighted that long-term survival in HGSC is more frequently observed in younger patients; however, this association is closely linked to other favorable features, including earlier stage at diagnosis, absence of ascites, and optimal cytoreduction [66]. Conversely, some studies suggest that the prognostic impact of age may be less pronounced when adjusted for key clinical variables. Baum et al. demonstrated that, after controlling for factors such as FIGO stage and residual disease, long-term survival was primarily associated with platinum sensitivity, absence of recurrence, and lower tumor burden, rather than age alone [67]. These findings indicate that age may act as a surrogate marker reflecting differences in treatment tolerance, performance status, and tumor biology, rather than serving as an independent determinant of outcome. The association between younger age and improved PFS is likely multifactorial. Younger patients generally have better performance status, tolerate platinum-based chemotherapy more effectively, and are more likely to undergo extensive cytoreductive surgery, while also potentially exhibiting more favorable tumor biology. Furthermore, aging is associated with immunosenescence, characterized by impaired T-cell function, reduced immune surveillance, and chronic low-grade inflammation, all of which may contribute to reduced treatment efficacy and earlier disease progression [65,68].

Regarding the progression-free survival analysis, the initial categorization of itTILs into three groups (<1%, 1–10%, and >10%) was pre-specified based on previously published methodology and biologically meaningful thresholds. Although this three-category analysis did not reach statistical significance (*p* = 0.102), the similarity of survival outcomes between the <1% and 1–10% groups prompted a post hoc exploratory analysis in which these two categories were combined and compared with the >10% group. This exploratory regrouping yielded that PFS in the itTIL > 10 group was 22 months longer than in the itTIL ≤ 10 group, which is statistically significant (*p* = 0.034), and in the multivariable Cox regression analysis, iTIL > 10 remained an independent predictor of shorter PFS (*p* = 0.045). However, we explicitly stated that this analysis was performed post hoc and should therefore be interpreted with caution. While the finding may represent a true biological effect, it may also reflect the impact of multiple testing and the relatively small number of patients in the itTIL > 10% subgroup (*n* = 20). Accordingly, this result should be considered hypothesis-generating and requires validation in larger independent cohorts.

Many studies assessing intraepithelial tumor-infiltrating lymphocytes (itTILs) use different quantification methods (number/HPF, density per mm^2^, median- or ROC-derived thresholds), and the authors themselves emphasize that no standardized cut-off has yet been established.

The association between high itTIL levels and prolonged PFS may reflect the biological importance of direct contact between cytotoxic lymphocytes and malignant epithelial cells. Unlike stromal lymphocytes, intraepithelial TILs are positioned to recognize tumor antigens presented by MHC class I molecules and mediate direct tumor cell killing. This spatial proximity may therefore better reflect effective immune surveillance than the overall quantity of immune cells within the tumor microenvironment [38,48,69]. All those findings support the idea that certain morphological patterns reflect underlying immune dynamics, which may have clinical significance in prognostic modeling and patient selection for targeted therapies. Studies investigating structural features of tumors in relation to immune microenvironment confirm that histopathological variations reflect essential biological events in the TME [70].

Several limitations of this study should be acknowledged. First, owing to its retrospective design, functional characterization of immune infiltrates was not feasible. Histological assessment and immunohistochemistry used provide information regarding the quantity and spatial distribution of immune cells but cannot distinguish activated cytotoxic lymphocytes from exhausted or functionally suppressed T cells. Second, regulatory T cells (Tregs) were not quantified. Previous studies have demonstrated that the CD8^+^/Treg ratio represents a more robust indicator of effective antitumor immunity than CD8^+^ cell density alone [38,71,72]. Consequently, although SET tumors demonstrated significantly higher CD8^+^ infiltration, we cannot determine whether these lymphocytes were present within an immunologically permissive or suppressive microenvironment. Future studies integrating functional immune markers and T-cell subset characterization will be required to clarify these mechanisms.

## 5. Conclusions

SET morphology in advanced-stage high-grade serous ovarian carcinoma is strongly associated with an immune-active tumor microenvironment characterized by increased stromal and intraepithelial tumor-infiltrating lymphocytes, higher CD8^+^ T-cell infiltration, increased PD-L1 expression, more frequent lymphoid aggregates, and a predominance of the inflamed immune phenotype. These findings indicate that SET morphology represents a readily identifiable histopathological surrogate of enhanced tumor immunogenicity.

Despite this pronounced immune activation, neither SET morphology nor most investigated immune parameters were independently associated with prolonged progression-free survival, suggesting that the presence of immune cells alone is insufficient to predict clinical outcome and may not accurately reflect their functional antitumor activity. In contrast, older age remained an independent predictor of shorter progression-free survival in the multivariable analysis, underscoring the importance of patient-related factors in determining clinical outcome. Although higher intraepithelial TIL levels were associated with improved progression-free survival in the univariable and multivariable analysis, their prognostic value requires further validation.

Overall, our findings suggest that routine histopathological assessment of SET morphology provides valuable insight into the immune landscape of HGSC and may facilitate future biomarker-driven patient stratification. Prospective studies integrating morphological assessment with functional immune profiling and molecular characterization are warranted to clarify the prognostic and predictive significance of these features and to optimize patient selection for immunotherapy.

## Figures and Tables

**Table 1:** Presents the percentages (%) of subjects for qualitative variables and the median (q1–q3, min–max) for quantitative variables based on SET morphology.

		SET Morphology				
Variable		No (*n* = 246)	Yes (*n* = 57)	*p* *	OR (95% CI)	*p* †
Age groups (years)				0.370		
	26–55 (*n* = 86)	68 (27.6)	18 (31.6)			
	56–61 (*n* = 67)	52 (21.1)	16 (28.1)			
	62–69 (*n* = 77)	63 (25.6)	14 (24.6)			
	>69 (*n* = 73)	63 (25.6)	9 (15.8)			
sTIL				<0.001	3.3 (1.9–5.6)	<0.001
	<1	32 (13)	1 (1.8)			
	1–10	161 (65.4)	28 (49.1)			
	>10	53 (21.5)	28 (49.1)			
itTIL				<0.001	4.8 (2.6–8.9)	<0.001
	<1	92 (37.4)	8 (14)			
	1–10	148 (60.2)	35 (61.4)			
	>10	6 (2.4)	14 (24.6)			
itTIL				<0.001	5.5 (3–10)	<0.001
	≤1	168 (68)	16 (28)			
	>1	78 (32)	41 (72)			
sCD8				0.006	2.4 (1.3–4.6)	0.005
	≤38.5	125 (51)	17 (30)			
	>38.5	120 (49)	40 (70)			
itCD8				0.001	2.9 (1.6–5.5)	0.001
	≤72.5	136 (56)	17 (30)			
	>72.5	109 (44)	40 (70)			
PD-L1 TPS				<0.001	4.7 (2.4–9.4)	<0.001
	<1	220 (90)	37 (65)			
	≥1	25 (10)	20 (35)			
PD-L1 CPS				<0.001	3.3 (1.8–5,9)	<0.001
	<1	189 (77)	29 (51)			
	≥1	56 (23)	28 (49)			
Primary LAs				0.017	2.3 (1.2–4.4)	0.012
	No	205 (83)	39 (68)			
	Yes	41 (17)	18 (32)			
Secondary LAs				0.007	4.8 (1.6–14)	0.005
	No	239 (97)	50 (88)			
	Yes	7 (3)	7 (12)			
Primary LAs/Secondary LAs				0.004	2.1 (1.3–3.3)	0.002
	No/No	205 (83)	39 (68.4)			
	Yes/No	34 (14)	11 (19.3)			
	Yes/Yes	7 (3)	7 (12.3)			
Tumor immunophenotype				<0.001	3.6 (2.3–5.5)	<0.001
	Desert	113 (45.9)	8 (14)			
	Excluded	69 (28)	6 (10.5)		1.2 (0.4–3.7)	0.714
	Inflamed	64 (26)	43 (75.4)		9.5 (4–21)	<0.001

* χ^2^ test; † odds ratio; abbreviations: SET—solid, pseudoendometrioid, and transitional cell-like morphology; sTIL—stromal tumor-infiltrating lymphocytes; itTIL—intraepithelial tumor-infiltrating lymphocytes; sCD8—stromal CD8^+^ lymphocytes; itCD8—intraepithelial CD8^+^ lymphocytes; PD-L1—programmed death-ligand 1; TPS—tumor proportion score; CPS—combined positive score; LAs—lymphoid aggregates.

**Table 2:** Correlation between progression-free survival (PFS) and the analyzed parameters, assessed using the log-rank test and Cox regression analysis.

Variable	Average (SE) 95% CI	Median (SE) 95% CI	LR; *p* ††	HR 95% CI	*p* *
SET morphology			2.6; 0.105	0.73 0.49–1.1	0.118
No	19.3 (2) 15–23	13 (0.7) 12–14			
Yes	24 (4) 16–36	16 (1) 13–19			
Age groups (years)			7.8; 0.049	1.2 1–1.3	0.020
26–55 †	28 (5) 19–37	15 (2) 10–20			
56–61	17 (2) 12–22	13 (1) 10–16			
62–69	17 (1) 14–20	13 (1) 11–15			
>69	16 (2) 11–21	12 (2) 8–15			
Age groups (years)			7.8; 0.005	1.5 1.1–2.1	0.007
26–55 †	28 (5) 19–37	15 (2) 10–20			
≥56	17 (1) 14–19	13 (0.8) 11–15			
sTIL			0.88; 0.644	0.97 0.75–1.2	0.828
<1	16 (3) 11–21	12 (2) 7–16			
1–10	22 (3) 17–27	14 (0.8) 12–15			
>10 †	18 (2) 14–21	13 (1) 10–16			
itTIL			4.6; 0.102	0.87 0.68–1.11	0.271
<1	18 (1) 15–21	14 (1) 11–16			
1–10	22 (2) 15–25	13 (0.9) 11–15			
>10	41 (13) 14–67	24 (18) 0–60			
itTIL			4.5; 0.034	2 1–4	0.043
≤10	19 (2) 16–23	13 (0.7) 12–14			
>10 †	41 (13) 14–67	24 (18) 0–60			
sCD8			0.075; 0.784	0.96 0.7–1.3	0.791
≤38.5	21 (3) 15–27	13 (1) 11–15			
>38.5	20 (2) 16–23	14 (1) 12–16			
itCD8			0.106; 0.745	0.95 0.71–1.3	0.753
≤72.5	18 (1) 16–21	14 (0.9) 12–16			
>72.5	22 (3) 16–29	13 (1) 11–15			
PD-L1 TPS			2.2; 0.14	1.4 0.9–2.3	0.156
<1	21 (2) 17–25	14 (0.7) 13–15			
≥1 †	15 (2) 11–19	10 (0.8) 8–12			
PD-L1 CPS			0.066; 0.797	0.96 0.7–1.3	0.803
<1	20 (2) 16–25	13 (0.7) 11–14			
≥1 †	20 (2) 15–25	14 (3) 9–19			

† Reference level; †† log-rank test; * Cox regression; abbreviations: PFS—progression-free survival; SET—solid, pseudoendometrioid, and transitional cell-like morphology; sTIL—stromal tumor-infiltrating lymphocytes; itTIL—intraepithelial tumor-infiltrating lymphocytes; sCD8—stromal CD8^+^ lymphocytes; itCD8—intraepithelial CD8^+^ lymphocytes; PD-L1—programmed death-ligand 1; TPS—tumor proportion score; CPS—combined positive score.

**Table 3:** Correlation between progression-free survival (PFS) and the analyzed parameters, assessed using the log-rank test and Cox regression analysis.

Variable	Average (SE) 95% CI	Median (SE) 95% CI	LR; *p* ††	HR 95% CI	*p* *
Primary LAs			0.142; 0.706	1.1 0.7–1.5	0.715
No	21 (2) 17–25	13 (0.7) 11–14			
Yes	17 (2) 14–21	15 (1) 13–17			
Secondary LAs			0.274; 0.600	1.2 0.6–2.4	0.614
No	20 (2) 17–24	13 (0.7) 11–14			
Yes	15 (3) 9–22	15 (3) 8–22			
Primary LAs/Secondary LAs			0.31; 0.857	1.1 0.81–1.4	0.633
No/No	21 (2) 17–25	13 (0.7) 11–14			
Yes/No	18 (2) 14–22	14 (1.3) 11–17			
Yes/Yes	15 (3) 9–22	15 (3) 8–22			
Tumor immunophenotype			2; 0.359		
Desert	20 (3) 14–26	14 (1) 11–17			
Excluded	17 (2) 13–21	13 (0.8) 11–14			
Inflamed †	23 (3) 16–29	14 (2) 10–17			

† Reference level; †† log-rank test; * Cox regression; abbreviations: LAs—lymphoid aggregates.

**Table 4:** Results of the multivariable Cox proportional hazards regression analysis assessing the association between progression-free survival (PFS) and the investigated variables.

Variable		HR	95% CI	*p*
Age groups (years)	26–55 †; >55	1.5	1.1–2.1	0.012
sTIL	<1 1–10 >10 †	0.99	0.75–1.3	0.920
itTIL	≤10 >10 †	2.1	1–4.4	0.045
PD-L1 TPS	<1 ≥1 †	1.65	1–2.7	0.051

† Reference level; abbreviations: sTIL—stromal tumor-infiltrating lymphocytes; itTIL—intraepithelial tumor-infiltrating lymphocytes; PD-L1—programmed death-ligand 1; TPS—tumor proportion score.

## Data Availability

The original contributions presented in this study are included in the article. Further inquiries can be directed to the corresponding author.

## Abbreviations

HGSC	High-Grade Serous Carcinoma
CA-125	Cancer Antigen 125
SET	Solid, Endometrioid, and Transitional-like
OS	Overall Survival
PARP	Poly(ADP-ribose) Polymerase
ICI	Immune Checkpoint Inhibitor
TME	Tumor Microenvironment
TILs	Tumor-Infiltrating Lymphocytes
TLS	Tertiary Lymphoid Structures
itTILs	Intratumoral Tumor-Infiltrating Lymphocytes
CD8	Cluster of Differentiation 8
FDA	Food and Drug Administration
PFS	Progression-Free Survival
PD-L1 CPS	Programmed Death-Ligand 1 Combined Positive Score
FIGO	International Federation of Gynecology and Obstetrics
RECIST	Response Evaluation Criteria in Solid Tumors
WHO	World Health Organization
HE	Hematoxylin and Eosin
LA	Lymphocyte Aggregate
GC	Germinal Center
PD-L1 TPS	Programmed Death-Ligand 1 Tumor Proportion Score
BRCA	Breast Cancer Gene
MHC	Major Histocompatibility Complex
TNBC	Triple-Negative Breast Cancer
TTP	Time to Progression
